# LS^X^: automated reduction of gene-specific lineage evolutionary rate heterogeneity for multi-gene phylogeny inference

**DOI:** 10.1186/s12859-019-3020-1

**Published:** 2019-08-13

**Authors:** Carlos J. Rivera-Rivera, Juan I. Montoya-Burgos

**Affiliations:** 1 Department of Genetics and Evolution, University of Geneva, Quai Ernest-Ansermet 30, 1211 Geneva, Switzerland; 2 Institute of Genetics and Genomics in Geneva (iGE3), University of Geneva Medical School, Rue Michel-Servet 1, 1211 Geneva, Switzerland

**Keywords:** Long branch attraction, Lineage rate heterogeneity, Phylogenomics, Phylogenetic methods, Sequence subsampling

## Abstract

**Background:**

Lineage rate heterogeneity can be a major source of bias, especially in multi-gene phylogeny inference. We had previously tackled this issue by developing LS^3^, a data subselection algorithm that, by removing fast-evolving sequences in a gene-specific manner, identifies subsets of sequences that evolve at a relatively homogeneous rate. However, this algorithm had two major shortcomings: (*i*) it was automated and published as a set of bash scripts, and hence was Linux-specific, and not user friendly, and (*ii*) it could result in very stringent sequence subselection when extremely slow-evolving sequences were present.

**Results:**

We address these challenges and produce a new, platform-independent program, LS^X^, written in R, which includes a reprogrammed version of the original LS^3^ algorithm and has added features to make better lineage rate calculations. In addition, we developed and included an alternative version of the algorithm, LS^4^, which reduces lineage rate heterogeneity by detecting sequences that evolve too fast *and* sequences that evolve too slow, resulting in less stringent data subselection when extremely slow-evolving sequences are present. The efficiency of LS^X^ and of LS^4^ with datasets with extremely slow-evolving sequences is demonstrated with simulated data, and by the resolution of a contentious node in the catfish phylogeny that was affected by an unusually high lineage rate heterogeneity in the dataset.

**Conclusions:**

LS^X^ is a new bioinformatic tool, with an accessible code, and with which the effect of lineage rate heterogeneity can be explored in gene sequence datasets of virtually any size. In addition, the two modalities of the sequence subsampling algorithm included, LS^3^ and LS^4^, allow the user to optimize the amount of non-phylogenetic signal removed while keeping a maximum of phylogenetic signal.

**Electronic supplementary material:**

The online version of this article (10.1186/s12859-019-3020-1) contains supplementary material, which is available to authorized users.

## Background

We recently showed that biases emerging from evolutionary rate heterogeneity among lineages in multi-gene phylogenies can be reduced with a sequence data-subselection algorithm to the point of uncovering the true phylogenetic signal [1]. In that study, we presented an algorithm called Locus Specific Sequence Subsampling (LS^3^), which reduces lineage evolutionary rate heterogeneity gene-by-gene in multi-gene datasets. LS^3^ implements a likelihood ratio test (LRT) [2] between a model that assumes equal rates of evolution among all ingroup lineages (single rate model) and another that allows three user-defined ingroup lineages to have independent rates of evolution (multiple rates model). If the multiple rates model fits the data significantly better than the single rate model, the fastest-evolving sequence, as determined by its sum-of-branch length from root to tip (SBL), is removed, and the reduced dataset is tested again with the LRT. This is iterated until a set of sequences is found whose lineage evolutionary rates can be explained equally well by the single rate or the multiple rates model. Gene datasets that never reached this point as well as the fast-evolving sequences removed from other gene alignments are flagged as potentially problematic [1]. LS^3^ effectively reduced long branch attraction (LBA) artifacts in simulated and biological multi-gene datasets, and its utility to reduce phylogenetic biases has been recognized by several authors [3, 4].

The published LS^3^ algorithm is executed by a set of Linux-specific bash scripts (“LS^3^-bash”). Here we present a new, re-written program which is much faster, more user-friendly, contains important new features, and can be used across all platforms. We also developed and included a new data subselection algorithm based on LS^3^, called “LS^3^ *supplement*” or LS^4^, which leads to lineage evolutionary rate homogeneity by removing sequences that evolve too fast and also those that evolve too slowly.

## Implementation

The new program, LS^X^, is entirely written in R [5], and uses PAML [6] and the R packages *ape* [7, 8] and *adephylo* [9]. If PAML, R, and the R packages *ape* and *adephylo* are installed and functional, LS^X^ runs regardless of the platform, with all parameters given in a single raw text control file. LS^X^ reads sequence alignments in PHYLIP format and produces, for each gene, a version of the alignment with homogenized lineage evolutionary rates. In the new program LS^X^, the best model of sequence evolution can be given for each gene, thus improving branch length estimations, and users can select more than three lineages of interest (LOIs) for the lineage evolutionary rate heterogeneity test (Additional file 1: Figure S1a,b).

Within LS^X^ we also implemented LS^4^, a new data subselection algorithm optimized for datasets in which sequences that evolve too fast and sequences that evolve too slow disrupt lineage rate heterogeneity. In such cases, the approach of LS^3^, which removes only fast-evolving sequences, can lead to the excessive flagging of data (Additional file 1: Table S1). This is because it will flag and remove sequences with intermediate evolutionary rates because they are still evolving “too fast” relative to the extremely slow-evolving ones (Additional file 1: Figure S2).

LS^4^ employs a different criterion to homogenize lineage evolutionary rates, which considers both markedly fast- and slow-evolving sequences for removal. Under LS^4^, when the SBLs for all ingroup sequences of a given gene are calculated, they are grouped by the user-defined LOI to which they belong. The slowest-evolving sequence of each LOIs is identified, and then the fastest-evolving among them across all ingroup lineages is picked as a benchmark (i.e. “the fastest of the slowest”, see Additional file 1: Figure S1c). Because in both LS^3^ and LS^4^ each LOI has to be represented by at least one sequence, this “fastest (longest) of the slowest (shortest)” sequence represents the slowest evolutionary rate at which all lineages could converge. Then, LS^4^ removes the ingroup sequence that produces the tip furthest from the benchmark, be it faster- or slower-evolving (Additional file 1: Figure S1d).

## Results

We compared the efficiency of LS^X^ relative to our previous script LS^3^-bash with simulated data (Additional file 1: Supplementary Methods), and found LS^X^ to perform the LS^3^ algorithm 7× times faster than LS^3^-bash with a 100-gene dataset, and 8× faster with a 500-gene dataset (Additional file 1: Table S1). We then compared the relative effectiveness of LS^4^ and LS^3^ when analyzing datasets in which there were mainly average- and fast-evolving sequences, and datasets in which there were very slow-, average-, and very fast-evolving sequences (Additional file 1: Supplementary Methods). In the former case, both LS^3^ and LS^4^ gave similar results (Additional file 1: Table S1). In the latter case, which includes very slow and very fast-evolving sequences, the data subsampling under LS^3^ was too stringent and reduced substantially the phylogenetic signal, and only the data remaining after LS^4^ were able to clearly solve the phylogeny (Additional file 1: Table S1). In addition, we applied both algorithms, as implemented in LS^X^, to a biological case study: a 10-gene dataset of the catfish order Siluriformes [10]. There are two conflicting hypotheses for the most basal splits of this phylogeny: one proposed by morphological phylogenetics, and one proposed by molecular phylogenetics (e.g. [11, 12]). The point of conflict is the positioning of the fast evolving lineage Loricarioidei, which is closer to the root in molecular phylogenies than in the morphological phylogenies. The attraction of the fast evolving Loricarioidei lineage towards the root may be an artifact due to strong lineage rate heterogeneity, and allowed us to explicitly test the different approaches of LS^3^ and LS^4^.

## Discussion

The results presented in [10] show that LS^3^ was able to find taxa subsets with lineage rate homogeneity in six out of the ten genes, and flagged four complete genes as unsuitable for analysis. Analyzing the LS^3^-processed dataset showed that the basal split of Siluriformes is indeed affected by lineage rate heterogeneity, and that there was a strong signal supporting the morphological hypothesis of the root. However, these results were not entirely satisfactory because one ingroup species was incorrectly placed among the outgroups, and one of the well-established clades of the phylogeny was not recovered. In contrast, LS^4^ found lineage rate homogeneity in seven out of the ten genes (only three genes were flagged), the final phylogeny showed the morphological hypothesis of the root, and all the ingroup taxa plus the well-established clades were recovered. In this case study, both LS^3^ and LS^4^ successfully mitigated the effect of lineage rate heterogeneity, but the data subselection criterion of LS^4^ allowed the inclusion of more data for the final analysis, and resulted in a phylogeny with better resolution.

## Conclusions

The new program presented here, LS^X^, represents a substantial improvement over our initial scripts in LS^3^-bash. LS^X^ is faster, platform-independent, the code is accessible, and also includes a new version of the algorithm, LS^4^. We show here and in a recent publication that this new version is more effective than LS^3^ in increasing the phylogenetic to non-phylogenetic signal ratio when extremely slow-evolving sequences are present in addition to very fast-evolving ones, and helped to solve a long-standing controversy of catfish phylogenetics. We also see a potential in both algorithms for scanning genome-wide datasets and using the gene flagging data to identify regions in which a single lineage shows a markedly accelerated evolution (such as human accelerated regions [13, 14]). Alternatively, the same data could also be used to identify genomic regions that are highly conserved (and thus slow-evolving) among some lineages but not others (e.g., conserved non-coding elements [15]). As research in phylogenetics progresses in the wake of the genomic era, we must begin to solve the most contentious nodes of the tree of life, where the usual methods may not be as effective. For undertaking these challenges we believe that accessible data subselection programs with clear criteria are a necessary tool, and should be made available whenever possible.

## Availability and requirements

**Project name:** LS^X^ v1.1.

**Project homepage:** https://github.com/carlosj-rr/LSx

**Operating systems:** Platform independent.

**Programming language:** R.

**Other requirements:** R 3.3.x or higher, R package *ape* 5.1 or higher (and dependencies), R package *adephylo* 1.1 or higher (and dependencies), PAML 4.

**License:** GNU GPL 3.0.

**Any restrictions to use by non-academics:** license needed.

## Additional file

Additional file 1: Supplementary Data. (DOCX 482 kb)

## Data Availability

LSx.R, the LS^X^ manual wiki, and example datasets are available at: https://github.com/carlosj-rr/LSx.

## Abbreviations

LBA: Long branch attraction
LOI: Lineages of interest
LRT: Likelihood ratio test
LS^3^: Locus specific sequence subsampling
LS^4^: LS^3^ supplement
SBL: Sum of branch lengths
