# Supplementary material for: LSX: automated reduction of gene-specific lineage evolutionary rate heterogeneity for multi-gene phylogeny inference
Source: BMC Bioinformatics. 2019 Aug 13;20:420. doi: 10.1186/s12859-019-3020-1 (PMC6693147; doi:10.1186/s12859-019-3020-1)
Supplement: Supplementary file 1 — Supplementary Data. (DOCX 482 kb) [file 12859_2019_3020_MOESM1_ESM.docx]

Supplementary Data

**LS^X^: Automated reduction of gene-specific lineage evolutionary rate heterogeneity for multi-gene phylogeny inference**

**Carlos J. Rivera-Rivera**^a,b^ and Juan I. Montoya-Burgos^a,b^*

^a^ Department of Genetics and Evolution, University of Geneva, Switzerland

^b^ Institute of genetics and genomics in Geneva (iGE3), University of Geneva, Switzerland

*** Corresponding author:** [**juan.montoya@unige.ch**](mailto:juan.montoya@unige.ch)

Contents:

[**Supplementary Methods**](#suppmeths)

[**Supplementary Table 1**](#supptab)

[**Supplementary Figure 1**](#suppfig1)

[**Supplementary Figure 2**](#suppfig2)

**Supplementary Methods**

*Producing the simulated datasets*

In order to compare LS^X^ and our previous script LS³-bash, we analyzed a 100-gene and a 500-gene randomized subsample from the datasets available as part of the original publication of LS³-bash^1^ (https://genev.unige.ch/research/laboratory/Juan-Montoya). These data were simulated following an 18-taxon tree that has average- and fast-evolving sequences. Each gene is of 2,000 bp, and evolved according to the Jukes-Cantor model of sequence evolution, with 0.3 proportion of invariable sites and site rate heterogeneity following the gamma distribution with the alpha parameter set at 1, and eight rate categories^1^. We analyzed both the 100- and the 500-gene datasets under LS³-bash, and under LS³ in LS^X^, as well as under LS^4^ in LS^X^ (Supplementary Table 1).

In order to show the relative efficiency of LS^4^ over LS³ in keeping more data when extremely short branches were present, we used a similar simulation strategy as in the original LS³-bash publication, but also added extremely slow evolving sequences. In the simulation procedure, the tree had four outgroups and three ingroup clades of six species each, for a total of 22 taxa. Within the ingroup, two clades had each four fast-evolving taxa, and two taxa that were average-evolving, and the third ingroup clade had four slow-evolving taxa and two average-evolving ones. We rotated the identity of the average-evolving taxa within the ingroups so that there would not be a single taxon that was slow- or fast-evolving in all simulated genes. We simulated a 100- and a 500-gene dataset, of 1,000 bp each gene, also under the Jukes-Cantor model, with 0.3 probability of invariable sites and site rate heterogeneity following the gamma distribution with the alpha parameter set at 1, and eight rate categories. We then analyzed both the 100- and 500-gene datasets with LS³-bash, and LS³ and LS^4^ in LS^X^.^.^ We measured how much phylogenetic information was left after flagging by concatenating the unflagged data of each analysis, and doing a tree inference with IQTree v1.6.9^2^, and calculating node supports with their ultra-fast bootstrap^3^.

All simulations were carried out with INDELible v1.03^4^, and all analyses were ran sequentially in a Lenovo® ThinkPad™ X230 laptop with 7.5 GiB of RAM, four Intel® Core™ i5-3320M CPUs @ 2.60GHz, and an SD memory, running under 64-bit Ubuntu 18.04. The R version used during the analyses was 3.4.4^5^.

**References**

1. Rivera-Rivera CJ, Montoya-Burgos JI. LS^3^: A Method for Improving Phylogenomic Inferences When Evolutionary Rates Are Heterogeneous among Taxa. Mol Biol Evol. 2016;33:1625–34.
2. Nguyen LT, Schmidt HA, von Haeseler A, Minh BQ. IQ-TREE: A Fast and Effective Stochastic Algorithm for Estimating Maximum-Likelihood Phylogenies. Mol Biol Evol. 014; 32:268-74.
3. Minh BQ, Nguyen MAT, von Haeseler A. Ultrafast approximation for phylogenetic bootstrap. Mol Biol Evol. 2013;30:1188-95.
4. Fletcher W, Yang Z. INDELible: a flexible simulator of biological sequence evolution. Mol Biol Evol. 2009;26(8):1879-88.
5. R Core Team. R: A language and environment for statistical computing. R Foundation for Statistical Computing, Vienna, Austria. Available online at <https://www.R-project.org/>. 2018.

**Supplementary Table 1 –** Results of the benchmarking studies of LS³-bash, and LS³ and LS^4^ under LS^X^. The three methods were employed to analyze four simulated datasets: a 100- and a 500-gene dataset with fast-evolving taxa, and a 100- and a 500-gene dataset with both slow- and fast-evolving taxa. For all datasets, the analysis time was shorter when using LS^X^. When analyzing the datasets with only fast-evolving sequences, LS³ and LS^4^ perform similarly, flagging an average of ~3 sequences, and keeping enough phylogenetic signal to solve the monophylies of the ingroup nodes. However, when analyzing the datasets in which extremely short branches are also present, LS³ is too stringent, and flags the majority of the data, while LS^4^ is able to flag problematic sequences more specifically and, thus, keeps more of the phylogenetic signal. For the mean flagged sequences per gene; if an entire gene was flagged, all sequences of that gene were counted as flagged. All analyses were ran sequentially in a Lenovo® ThinkPad™ X230 laptop with 7.5 GiB of RAM, four Intel® Core™ i5-3320M CPUs @ 2.60GHz, and an SD memory, running under 64-bit Ubuntu 18.04.

**
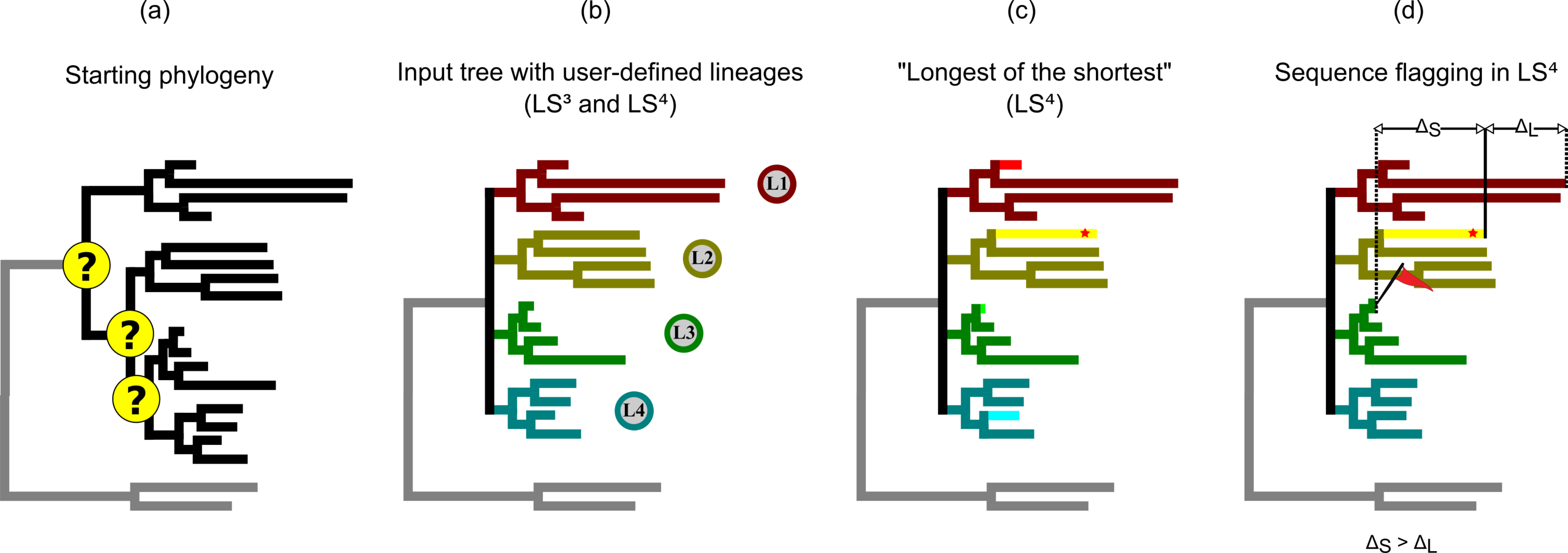
**

**Supplementary Figure 1.** A schematic representation of the procedure for flagging sequences in LS⁴. (*a*), The general phylogeny for this group of taxa, with the nodes in question highlighted. (*b*), For LS³ and LS⁴, an input tree is given in which the nodes into question are collapsed, and the lineages involved are identified . (*c*), LS⁴ identifies the slowest evolving sequence for each clade (highlighted), and then identifies the fastest evolving among them (red star). (*d*) The sequence to be removed in each iteration of LS⁴ is the one corresponding to the tip furthest from the tip of the “fastest of the slowest” branch, resulting in the flagging of sequences that evolve too fast as well as sequences that evolve too slow.

**
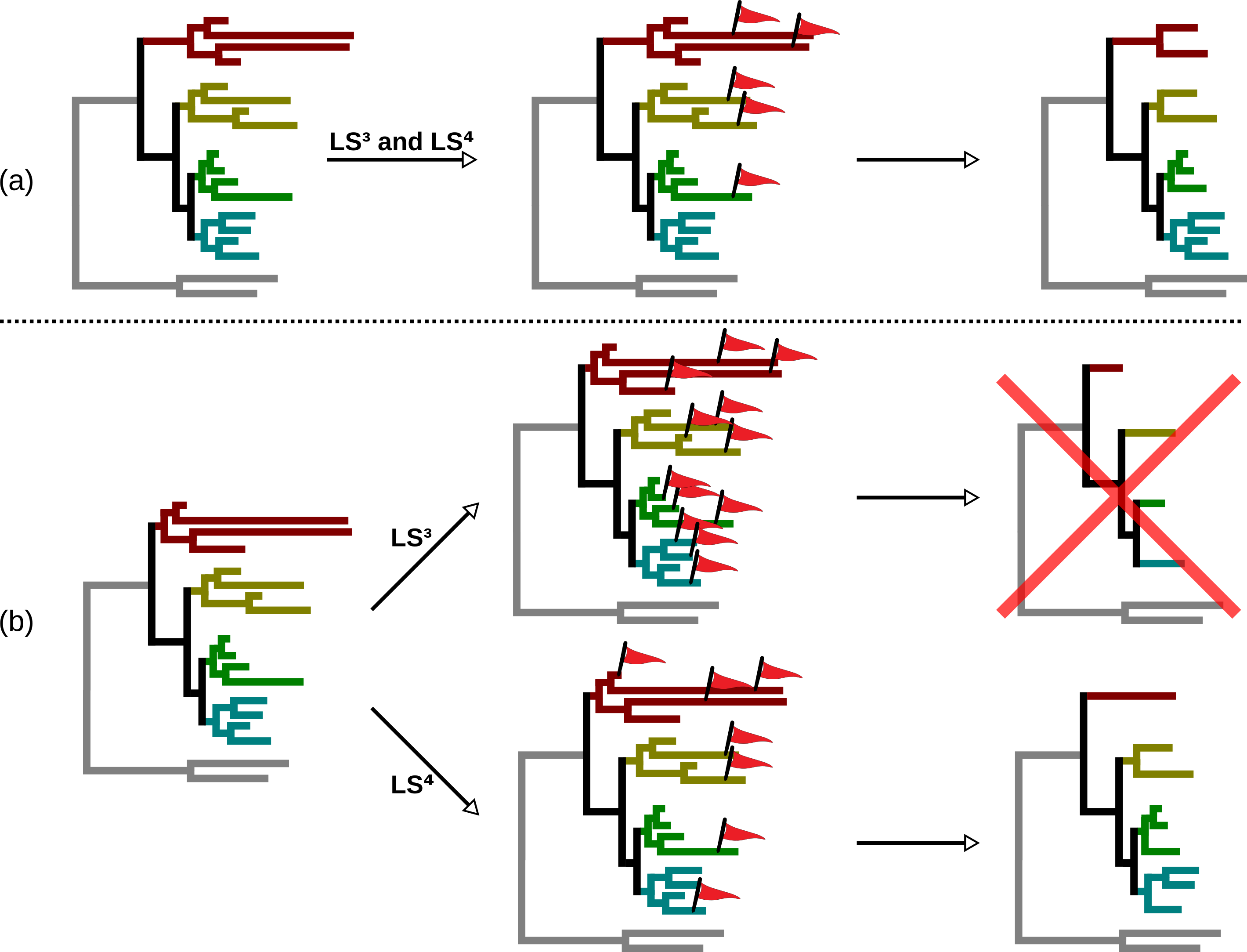
**

**Supplementary Figure 2.** A schematic representation of the different ways that LS³ and LS^4^ reach lineage rate homogeneity for a given gene sequence dataset. In (*a*), a dataset with a general rate homogeneity with the exception of several faster evolving branches. In this case, both methods will flag the faster evolving sequences, and reach the same taxon subset with homogeneous rates of evolution. In (*b*), a dataset with general lineage rate heterogeneity, and with a very short branch (the top branch of that tree). In such a case, LS³ will remove all of the faster evolving sequences, until only the slowest sequence of each clade of interest remains. At this point, that gene sequence dataset is flagged completely as problematic because lineage rate heterogeneity is still too strong. In contrast, LS^4^ will remove the faster evolving sequences and also the slowest one, thus reaching lineage rate homogeneity for this dataset.
